# Comparison of Anterior vs. Dorsal Approach for Spinal Accessory to Suprascapular Nerve Transfer in Patients With a Brachial Plexus Injury and Its Outcome on Shoulder Function

**DOI:** 10.7759/cureus.26543

**Published:** 2022-07-04

**Authors:** Hasan Tahir, Muhammed Osama, Mirza Shehab A Beg, Mehtab Ahmed

**Affiliations:** 1 Plastic Surgery, Liaquat National Hospital, Karachi, PAK; 2 Plastic and Reconstructive Surgery, Liaquat National Hospital and Medical College, Karachi, PAK

**Keywords:** shoulder function, nerve transfer, spinal accessory nerve, suprascapular nerve, dorsal approach, anterior approach, bilateral brachial plexus injury

## Abstract

Background

Brachial plexus injuries are frequently encountered in the domain of plastic surgery, mostly secondary to road traffic accidents, gunshot injuries, or falls from a height. Many modalities have been described in the management, depending on the level and duration of the injury. C5, C6 and C5, C6, C7 are two common patterns in which nerve repair and transfers are described. At our center, we practice spinal accessory to suprascapular nerve transfer in all patients with upper trunk brachial plexus injury. There are two described approaches for the spinal accessory nerve to suprascapular nerve transfer, i.e. anterior or dorsal. The rationale for doing the posterior approach is that this approach avoids damaging the suprascapular nerve at its entrance in the suprascapular notch under the suprascapular ligament during exploration due to traction.

Materials and methods

This is a retrospective study with a consecutive sampling of 23 patients presenting at Liaquat National Hospital, Karachi, with upper trunk brachial plexus injuries during the time period from January 2016 to December 2017, i.e. two years. We divided these 23 patients into two groups, one with the anterior approach and the other with a dorsal approach for spinal accessory to suprascapular nerve transfer for shoulder abduction. The mean duration of post-surgical follow-up was from 18 to 24 months and recovery and functional outcomes were assessed.

Results

Out of the 23 patients that were included, 10 patients were operated on with an anterior approach and 13 with a posterior approach. Fifty percent (50%) of patients operated with the anterior approach and 84% of patients with the posterior showed the best motor grade recovery of M4, respectively, with better performance in patients with the posterior approach as compared to the anterior approach.

Conclusion

We advocate taking a posterior approach for spinal accessory to suprascapular nerve transfer for shoulder abduction, as it has shown better results with reliable outcomes concerning shoulder abduction, angle of abduction, and range of motion.

## Introduction

Brachial plexus injuries are commonly seen secondary to birth-related trauma or from trauma secondary to a fall, most commonly in a road traffic accident [1-4]. Injuries involving the upper trunk brachial plexus, i.e., C5, C6, C7 roots, lead to significant disability of shoulder function with loss of shoulder abduction and range of motion. This devastating injury leads to loss of shoulder and elbow function thereby leaving the victim with a significant disability [5]. There is restricted/absent/weak shoulder abduction and external rotation [6]. Restoration of form and function remains the main aim of the surgeon in dealing with such injuries. In root avulsion injuries, no donor stumps are available for repair and nerve transfer remains the favorable option [7]. For restoration of shoulder function, different nerve transfers have been described in the literature [7-12].

The most commonly and widely used donor is to spinal accessory nerve [13]. Others use the phrenic nerve as the donor to the suprascapular nerve [14]. The action of the spinal accessory nerve on the trapezius in shoulder elevation is synergistic with shoulder abduction, therefore, it has been described by authors as the target donor nerve to the suprascapular nerve [15]. There are two described approaches for the spinal accessory nerve to suprascapular nerve transfer, i.e. anterior or dorsal [16-17]. When approaching anteriorly, the nerves are explored anteriorly and transfer is performed via the same incision as that for brachial plexus exploration [16-19]. The rationale for utilizing the posterior approach is that during exploration, the chances of damaging the suprascapular nerve, as it enters the suprascapular notch under the suprascapular ligament due to traction are reduced and optimum results can be achieved [20-21]. This, however, cannot be achieved by an anterior approach where exploration of the distal course of the suprascapular nerve around the suprascapular notch is unattainable [21-23].

In the present study, the aim is to compare the postoperative outcome of the anterior vs. dorsal approach in patients undergoing spinal accessory to suprascapular nerve transfer in terms of shoulder abduction.

## Materials and methods

This is a retrospective cohort study with a consecutive sampling of 23 patients presenting with upper trunk brachial plexus injuries in the department of Plastic and Reconstructive Surgery, Liaquat National Hospital, Karachi, during the tenure extending from 1st January 2016 to 31st December 2017, i.e. two years.

All patients who have avulsion injuries of upper trunk brachial plexus at C5, C6 and C5, C6, C7 with no clinical signs of recovery at least three months post-injury were included in the study irrespective of age, gender, and occupation. Patients with prior brachial plexus exploration or nerve repair, either primary or graft performed, were excluded. The sample size was calculated using the G power sample size calculator. The effect side was taken as large (0.9), and the α error probability was taken as 0.05. Power (1-β error probability) was taken as 0.8 and the allocation ratio Group 1/Group 2 was taken as 0.7, as we have lesser cases operated for Group 1. Based on the above values, the total sample size was calculated to be 23 (Groups 1 - 10 and Groups 2 - 13).

**Figure 1:**
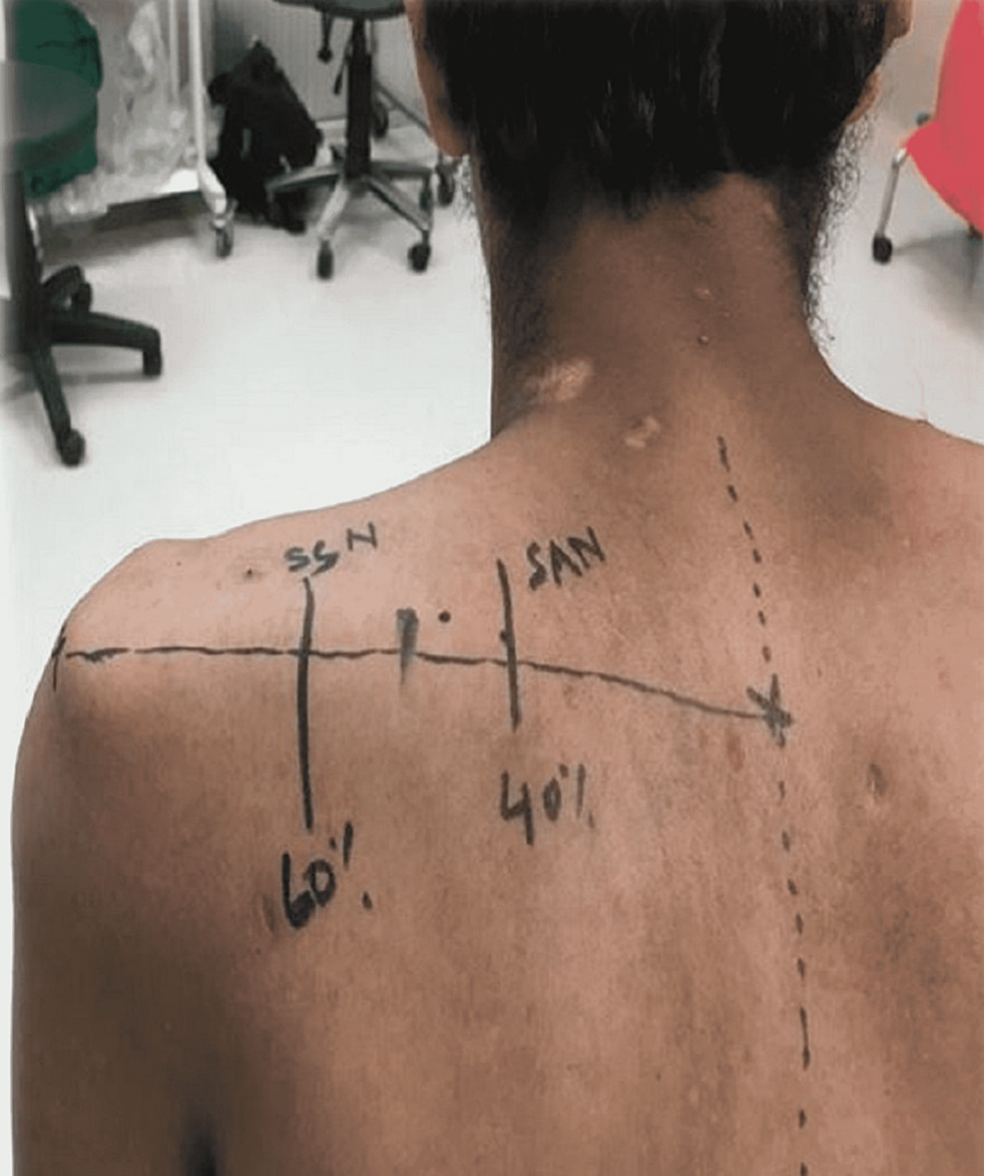
Surgical marking on the patient undergoing nerve transfer via the posterior approach

Patients were divided into two groups. In Group 1 patients, transfer was performed via the anterior approach, and Group 2 patients underwent nerve transfer via the posterior approach (Figure 1).

Data were collected using a proforma. The medical research council (MRC) grading system was used to assess functional outcomes at three months, six months, 12 months, and 18 months postoperatively [24].

Data were entered and analyzed via the SPSS version 25.0 statistical package (IBM Corp., Armonk, NY). Results are presented as mean + SD for continuous variables, i.e. age, and as frequency/percentage for nominal variables, i.e. gender, site, mechanism of injury, type of nerve repair, and signs of nerve recovery on electromyogram (EMG)-nerve conduction study (NCS). Pie and bar charts were utilized for the graphical display of results. Outcomes of both the procedures were compared using the Pearson chi-square and Fisher's exact tests.

## Results

**Figure 2:**
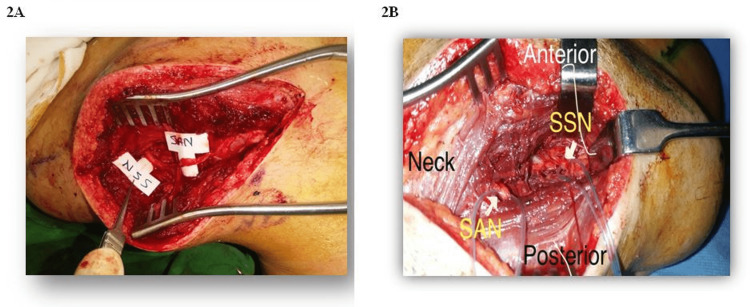
Intraoperative view of spinal accessory nerve and suprascapular nerve being identified and isolated

The study was conducted at Liaquat National Hospital from January 2015 to December 2016, i.e. two years. Twenty-three patients underwent spinal accessory to supra-scapular nerve transfer for shoulder abduction (Figure 2). All patients in our study were males with a mean age of 28.43 (SD 5.647).

All had avulsion injuries of the upper trunk brachial plexus at the C5, C6, and C5, C6, C7 levels with none having any signs of clinical improvement during the period of three months, with an average duration from injury to surgery of three to nine months. Patients were divided into anterior and posterior groups. Ten (43.5%) patients underwent nerve transfer via an anterior approach while 13 (56.5%) patients underwent nerve transfer via a posterior approach. The mean operative time was 4.5 hours in all the cases. The mean duration of post-surgical follow-up was from 18 to 24 months for assessment of recovery and functional outcome. Fortunately, all patients were compliant and came for a follow-up visit on advised dates.

**Figure 3:**
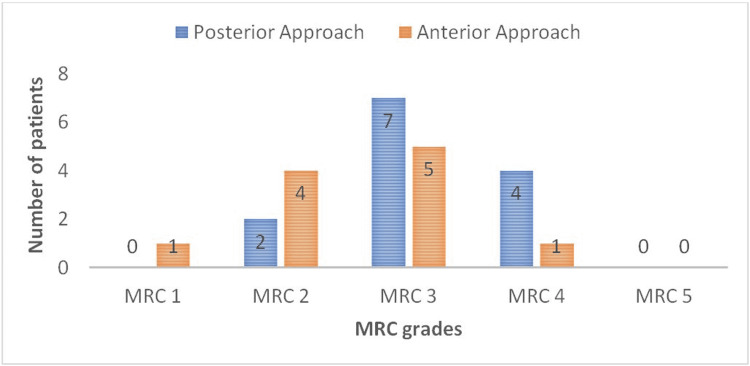
Demonstrates the MRC grades achieved by patients in respective groups MRC: medical research council

Improvement in MRC grades was documented on each follow-up visit. Maximum MRC grade achievement was MRC 4, which was seen at the 18 and 24-month follow-up visits with none achieving MRC grade 5 for shoulder abduction (Figure 3, Table 1).

**Table 1:** Demonstrates a comparison of the anterior and posterior approaches

Case	Gender	Age (years)	Approach taken	Follow-up time (months)	MRC grade
1	Male	21	anterior	24 months	4
2	Male	20	anterior	24 months	3
3	Male	22	anterior	24 months	3
4	Male	23	anterior	24 months	3
5	Male	22	anterior	24 months	3
6	Male	23	anterior	24 months	1
7	Male	25	anterior	24 months	2
8	Male	26	anterior	24 months	2
9	Male	27	anterior	24 months	2
10	Male	26	anterior	24 months	2
11	Male	30	posterior	24 months	4
12	Male	29	posterior	24 months	4
13	Male	28	posterior	24 months	4
14	Male	26	posterior	24 months	4
15	Male	28	posterior	24 months	3
16	Male	30	posterior	24 months	3
17	Male	35	posterior	24 months	3
18	Male	34	posterior	24 months	3
19	Male	33	posterior	24 months	3
20	Male	32	posterior	24 months	3
21	Male	35	posterior	24 months	3
22	Male	39	posterior	24 months	2
23	Male	40	posterior	24 months	2

**Figure 4:**
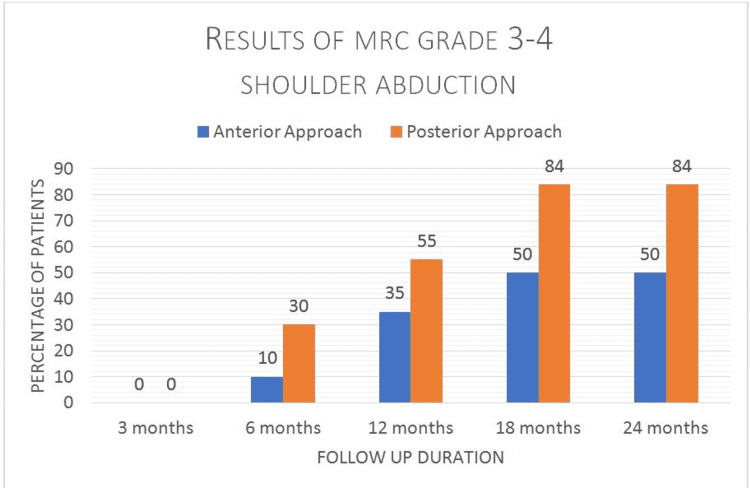
Demonstrates the improvement in MRC grades over the 03, 06, 12, 18, and 24 months follow-up for shoulder abduction in each group MRC: medical research council

From our study, we can see that only 50% of patients achieved MRC grades 3-4 with the anterior approach, whereas with the posterior approach, it is 84% (Figure 4).

Chi-square statistics were applied, which demonstrated a significant p-value (<0001) showing the significant difference and better results/functional outcomes with a posterior approach. MRC grades with each approach were compared, which showed the superiority of the anterior approach up to Grade 2 with the supremacy of the posterior approach and a better outcome for Grades 3 and 4.

## Discussion

Improving shoulder function after a brachial plexus injury is of utmost importance in rehabilitation after such drastic injuries. In our study, acceptable functional recovery and favorable results in terms of the shoulder were noted with the spinal accessory nerve to suprascapular nerve transfer, with better performance noted in the posterior approach as compared to the anterior approach. Results were assessed using the MRC scale for motor power assessment [25-27]. Eighty-four percent (84%) of patients achieved the best motor grade of 3-4 who underwent transfer via the dorsal approach as compared to only 50% of patients achieving the same grade via the anterior approach.

**Figure 5:**
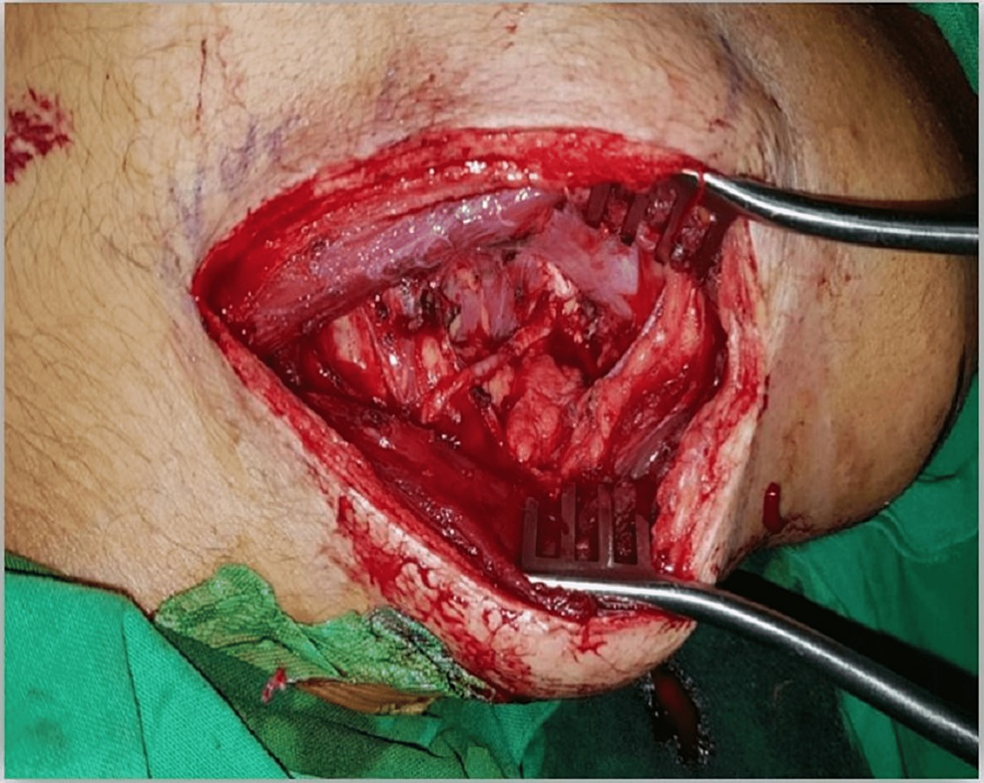
Intraoperative view of coaptation of the two nerves via the posterior approach

 We believe there are certain reasons for the dorsal approach to be superior in achieving such results. First, supply to the proximal trapezius muscle is preserved. Second, this approach also allows direct visualization of both nerves to be coapted (Figure 5) and, therefore, avoids chances of missing a double crush injury to the suprascapular nerve during exploration and yielding better functional improvement [13,28]. It also facilitates early recovery by the proximity of repair to the muscle and the location of the repair, protecting it from vigorous movement at the neck [28-30].

Teriz et al. reported that one of the three sites for potential damage to the suprascapular nerve is its course at the suprascapular notch [31]. This distal injury may lead to the double crush syndrome of the nerve and to poor outcomes in patients [32-33]. Having taken the anterior approach where distal nerve exploration is difficult [34], the chances of missing a double crush injury to the suprascapular nerve are higher and can be avoided while approaching nerves dorsally. We believe that better performance in group B patients may be partly due to taking into consideration distal exploration of the suprascapular nerve before coaptation.

A similar study conducted by Bhandari et al. on 14 patients reported the dorsal approach was better for nerve transfer with no distal injuries encountered in the suprascapular nerve [29]. In our study, we found similar results in patients with a posterior approach, making this approach a better option.

Like other studies, our study and approach of choice also have certain limitations. Considering the nature of data collection, it was a retrospective study and blinding was not done, which might have affected the study outcomes during evaluation on follow-up visits. Having to change the position of the patient intraoperatively is one limitation of the dorsal approach, which is avoidable in the anterior approach. The other limitation of the dorsal approach is a requirement of laborious traction, good illumination, and higher magnification during suprascapular nerve exploration due to its deep location in the suprascapular notch [35].

## Conclusions

As evident in the present study, it can be concluded that the posterior approach for spinal accessory to suprascapular nerve transfer for shoulder abduction in upper trunk brachial plexus palsy has better results, with reliable outcomes concerning shoulder abduction, angle of abduction, and range of motion.
